# Three Risky Decades: A Time for Econophysics? †

**DOI:** 10.3390/e24050627

**Published:** 2022-04-29

**Authors:** Ryszard Kutner, Christophe Schinckus, Harry Eugene Stanley

**Affiliations:** 1 Faculty of Physics, University of Warsaw, Pasteur Str. 5, PL-02093 Warsaw, Poland; 2 School of Business, University of the Fraser Valley, 33844 King Road, Abbotsford, BC V2S 7M8, Canada; chris.schinckus@ufv.ca; 3 Department of Physics, Boston University, 590 Commonwealth Ave, Boston, MA 02215, USA; hes@bu.edu

## 1. Motivation

The Special Issue comes out in the increasing accumulation of negative global tensions in many areas. The year 2022 seems the most unpredictable of the post-second world war years—a true sanitary/humanitary, climatic, and socio-economic thriller. Among these global challenges, two far-from-stationary (or unstable) phenomena and processes (operating at various spatio-temporal scales) need to be mentioned: (1) the pandemic shock and its economic effects [1], as well as the enormous social frustrations it generates, and (2) the climatic change that is progressing at an alarming pace, resulting in rapidly increasing migrations on a global scale. These aspects overlap, of course, with the tension between different cultures, religions, political systems, and the rivalry of superpowers. Finally, we must bear in mind the impact of local phenomena and processes (such as those caused by Brexit or the attack on the Capitol—both due to solid social polarization). To a greater or lesser extent, all of these are burdened by media information that is playing an increasing role in our society through its growing social impact. In such a context, characterized by extreme/rare and super-extreme events combined with tremendous volatility and giant fluctuations as well as the extraordinary ease of the spread of information and epidemics, our everyday life became more and more uncertain and even more turbulent—all aspects of our society are impacted by these global and local factors. All these, combined with the surprising helplessness of central banks and international financial institutions, result in the decline of the level of investment, an increase in unemployment, extraordinary involvement of states in the economy, inflation, and stagnation, and consequently, a recession. 

Given the long-term correlations, multiscale/multifractality, criticality, and complexity of the challenges mentioned above, an interdisciplinary science is essential to structure, understand and eventually predict the way our societies can evolve. By combining scientific methods that is, utilizing physics to study socio-economic realities, econophysics and sociophysics offer necessary interdisciplinary approaches. 

Increasing sanitary, climatic, and socio-economic uncertainty can take several forms and impact markets through various market bubbles and collapses (increasing all forms of risk). In this challenging time overwhelmed by information and data, an interdisciplinary approach such econophysics and sociophysics can be particularly useful in rationalizing and reducing the aforementioned risks. This Special Issue illustrates how the combination of scientific fields can provide fruitful conceptual frameworks to understand the current unprecedented transformation of our society.

In conjunction with a previous issue [2,3], this current Special Issue shows the multi-branch nature of econophysics and sociophysics topics and the diversity of econophysicists’ and sociophysicists’ interests, reflecting the diversity of the world around us. It pushes for not only a qualitative but, above all, quantitative description of reality from very different, complementary points of view. As a society, we are ready to acquire, collect, develop and publish empirical data, cause analysis, analysis of effects, analysis of mechanisms, and statistical forecasting and proposed actions. The condition is transparency/widespread availability of unadulterated empirical data collected by various independent institutions and portals. We have presented the content of our Special Issue in Sec. V below.

This Special Issue is published under the extraordinary situation—it is a testimony of the pre-current war world, bearing witness and summarizing the era that is just passing. Now we face the challenge of understanding and describing the world to come—the world in which globalization ties with the reevaluation of pre-current war paradigms. 

## 2. Remarks on Prehistory

Econophysics does not come from nowhere and it can be related to some early works developed by some: Louis Bachelier (LB) and especially Jan Tinbergen (JT). Although the former was an expert in mathematical physics, the latter was an active physicist.

**Figure 1:**
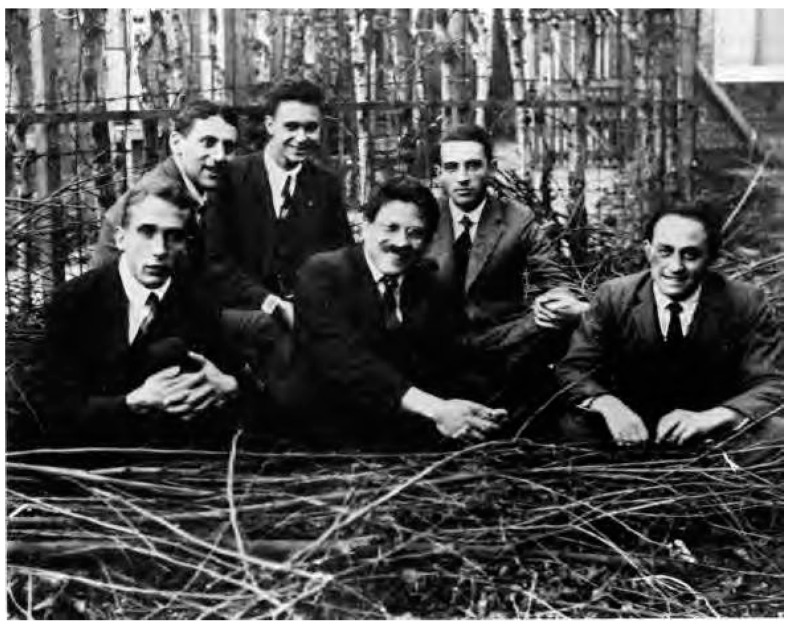
Group of Paul Ehrenfest students and friends (Leiden 1924). From the left to the right: Gerhard Dieke, Samuel Goudsmit, Jan Tinbergen, Paul Ehrenfest, Ralf Kronig, and Enrico Fermi. *Public photo was taken from the Internet*.

Louis Bachelier defended his doctoral dissertation in 1900 [4] under the supervision of Henri Poincaré—his research introduced the hypothesis on the stochastic nature of financial markets. It has been just 100 years since Jan Tinbergen began studying mathematics and physics at the University of Leiden (the Netherlands) with Paul Ehrenfest who appeared on the photo below (see Figure 1). 

In 1926 Jan Tinbergen graduated from university. In 1929 he defended his doctoral thesis entitled “Minimumproblemen in de natuurkunde en de economie” under the supervision of Paul Ehrenfest. This thesis is the first attempt in the intellectual history to combine natural and economic sciences through a strictly quantitative approach by using physics as theoretical reference. Jan Tinbergen’s work was directly influenced by his supervisor’s (Paul Ehrenfest) research interest including, among other things, the analogy between thermodynamic formalism and economic processes. Generally speaking, Tinbergen initiated the idea of using physics in economics. Jan Tinbergen was the first Nobel Prize laureate (which he received it with Radgar Frisch) in economics in 1969 and he is nowadays seen as the father of econometrics.

Bachelier and Tinbergen laid down the epistemological foundations for a more quantitative approach of the socio-economic reality. This path became gradually inspiring and generated a constant increase in interest, as illustrated below.

**Figure 2:**
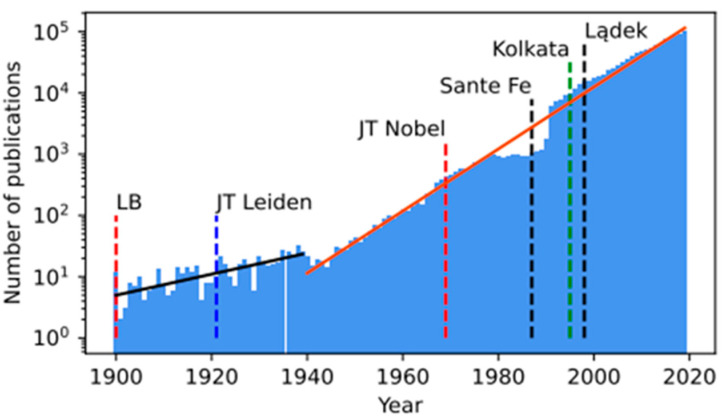
Depending on the time, the annual number of publications (NP) related to the use of physics methods in economics and finance. There are two different regions characterized by different values of the growth factor 1/τ. Namely, NP~exp (t/τ), where τ = 25.23 [Year] for the range 1900–1938, and τ = 8.58 [Year] for the range 1939–2019. We have marked the following events in the plot. “LB’’ (in 1900) marks the appearance of the doctoral dissertation (mentioned in the text) by Louis Bachelier, “JT Leiden’’ (year 1921) dates Jan Tinberger joining the University of Leiden, “JT Nobel’’ (year 1969) means the receipt of the Nobel Prize by Jan Tinbereger, “Santa Fe’’ marks the ground-breaking conference of the Santa Fe Institute (1987) mentioned in the text, “Kolkata’’ means the historic conference in Kolkata (India, 1995) and “Lądek’’ the conference in Lądek Zdrój (Poland, 1998)—both related significantly to econophysics. The last two conferences were the precursors of cyclical econophysics conferences held to this day: Econophysics Symposium (FENS in Poland since 2004) and Econophysics Colloquium (organized by Tiziana Di Matteo in various countries since 2005) as well as conferences organized in this century by Wei-Xing Zhou (East China University of Science and Technology) and Hideki Takayasu (Nikkei Institute, Sony, Tokyo, Japan). *The plot was made by Jarosław Klamut, the PhD-student of one of us (RK). The plot was published with his consent*.

Figure 2 shows a histogram for annual publications in the area of science that we call econophysics today. The plot was built on publications extracted using over 70 characteristic vital names and phrases from nearly 45 journals registered with Web of Science (WofS) database. 

The histogram begins in 1900, the year of the publication of the above-mentioned doctoral dissertation by Louis Bachelier [4]. The exponential growth of the histogram is divided into two time periods. The first period was from 1900 to 1938 and the second from the outbreak of World War II in 1939 to 2019. The growth rate of 1/τ for the first period is about three times lower than for the second period. Moreover, an approximately ten-year publication “gap” in the latter half of this later period is clearly visible. 

In 1987, in the very center of above-mentioned gap, a conference was held by the Santa Fe Institute, chaired by two Nobel Prize laureates: economist Kenneth Joseph Arrow—Nobel Prize in Economic Sciences (1972) together with John Hicks for their pioneering contributions to general economic equilibrium theory and welfare theory, and physicist Philip Warren Anderson—Nobel Prize in Physics (1977) together with Nevill Francis Mott and John Hasbrouck Van Vleck for their fundamental theoretical investigations of the electronic structure of magnetic and disordered systems. This pioneering conference aimed to answer the question: how economics can benefit from physics, computer science, and biology. This conference initiated a cascade of publications that continues to this day. We can formally treat the right slope of the publication gap shown in Figure 2, as the beginning of modern econophysics [5].

## 3. Remarks on History

There is a good reason for this Special Issue: the year that has just passed marked the third decade of a new way of dealing with economics through the lens of a physics-based approach on a large scale. Since then, there has been an increasing number of publications (included in the WofS database) devoted to what is now called econophysics. The origins of this movement are complex and manifold. A possible catalyst for this increase is the famous conference at the Santa Fe Institute in 1987, organized indeed by Kenneth Arrow and Philip Anderson. The latter was a co-founder of the Institute, which had been brought into being three years earlier. The mission of the Institute has been defined as „Searching for Order in the Complexity of Evolving Worlds”—the above-mentioned event fits perfectly into it.

The purpose of this event was to see how economics could benefit from physics, computer science, and biology. Econophysics may be related to the ground-breaking work (“Lévy walks and enhanced diffusion in Milan stock exchange”) written by the physicist Rosario N. Mantegna in Physica A (1991)—this article, considered by many to be the beginning of modern econophysics, showed that we had entered in an era of extreme and rare events as we experience it almost every day. In addition to these potential origins, other important works also contribute to the development of research related to econophysics: among others, one can quote, “Statistical properties of deterministic threshold elements—the case of market price” by H. Takayasu, H. Miura, T. Hirabayashi, K. Hamada in Physica A (1992), or “The Black-Scholes option pricing problem in mathematical finance: Generalization and extensions for a large class of stochastic processes” by J-P. Bouchaud and D. Sornette in J. Phys. I France (1994). We have just cited some of these works here, realizing that this is a subjective selection that reflects our point of view. In this Special Issue, all perspectives on econophysics are welcome, even though they might generate controversial discussions or opposite viewpoints. The authors will have the opportunity to put forth their way of presenting and working with econophysics.

The new era evoked above cannot be characterized through the classical Brownian and Gaussian behavior (Wiener process) originally discovered by Louis Bachelier in his dissertation [4]; instead, the statistical characterization of our contemporary world is more in line with a Lévy flight process over multiple timescales identified by Mantegna in his article on the Milan Index mentioned above. In this context, the central limit theorem has been replaced by the Lévy–Khintchine generalized central limit theorem. These findings have been confirmed by later works—see Mantegna-Stanley in Nature (Vol. 376(6), 1995). 

In a short period of time, an avalanche of publications created an apparently impossible bridge between physics and socio-economic sciences (especially financial markets). In this Special Issue, eminent scholars have been invited, all of whom have significantly contributed to econophysics. We hope their writings will illustrate and exemplify the history of econophysics, the current trends in the field, as well as its future perspectives. We voluntarily keep open the scope of this Issue leaving to the authors’ decision what they consider to be the milestones of econophysics and how they see its future. We want econophysics to be presented from different points of view, even though these views might be contradictory or sources of internal scientific tensions. Our work “Econophysics and sociophysics: Their milestones & challenges’’ in Physica A (2019) can be used as a source of inspiration for the celebration of the development of econophysics. As Guest Editors, we believe that the Special Issue will be scientifically attractive and inspiring. The 30th anniversary is in opportunity to show econophysics as a living and developing field of science related to many other fields. This Special Issue does not aim to be a museum but instead an inspiring collection of writings opening up prospects for the future of the field.

This Special Issue is also a way to present econophysics to the general public and to scholars who are external to the field: its achievements, its challenges, and even the controversial opinions/internal tensions and sometimes contradictions that might have emerged in the field. As Guest Editors, we are keen to show that econophysics is alive and inspiring—especially in the context of the global challenges with which we are faced.

## 4. Conclusions

**Figure 3:**
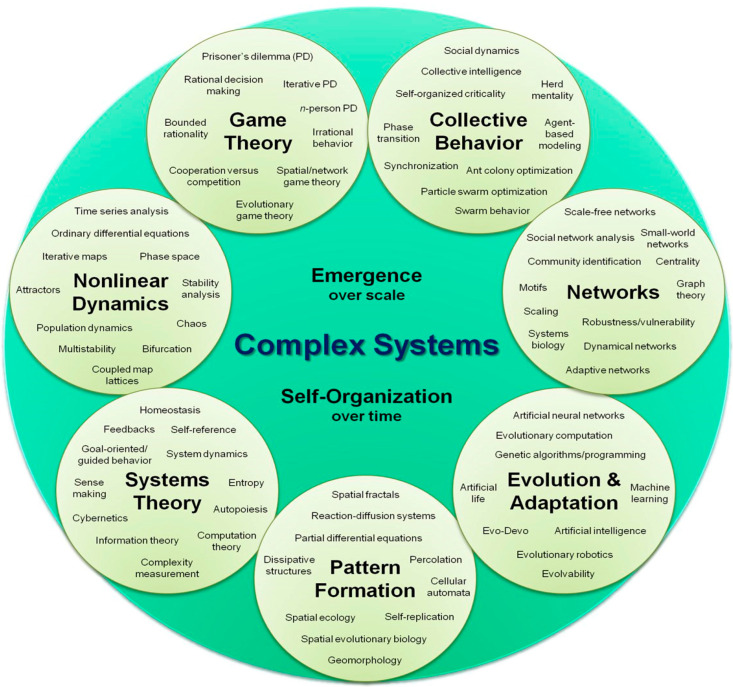
Schematic indication of the wealth of areas of the complex science with which econophysics and sociophysics are related. *Public drawing was taken from the Internet*.

We conclude this Editorial with an illustration (shown in Figure 3) characterizing the relationship between econophysics/sociophysics and the fields related to complexity. 

This Special Issue provides 33 articles, we have arranged them, for convenience, in 9 sections exemplifying these epistemic interactions. This arrangement is ambiguous because many works cover several research directions. 

However, it is impossible to frame the entire wealth of contemporary econophysics and sociophysics in a single Special Issue. Nevertheless, we hope that we present to the readers work containing new inspiring concepts and an overview of the crucial achievements of econophysics and sociophysics so far.

## 5. Content

As evoked above, econophysics has many connections with several subfields and this Special Issue aimed at capturing this intellectual richness. With this purpose, the content of this issue can be summarised as follows:

**i.**  **Econophysics as a Complex System: History, Economic Freedom, State of the art, and Econophysics Perspectives**

Economic freedom: The Top, the Bottom, and the Reality. I. 1997–2007 by *Marcel Ausloos and Philipe Broniet*

Plotting the Words of Econophysics *by Gianfranco Tusset*

Development of Econophysics: A Biased Account and Perspective from Kolkata *by Bikas K. Chakrabarti and Antika Sinha*

Radical Complexity *by Jean-Philippe Bouchaud*

Three Decades in Econophysics—From Microscopic Modelling to Macroscopic Complexity and Back *by Alex Smolyak and Shlomo Havlin*

Valuing the Future and Discounting in Random Environments: A Review *by Jaume Masoliver, Miquel Montero, Joseph Perello, J. Doyne Farmer, and John Geanakop*

**ii.**  **Time Series Analysis**

Relationship between Continuum of Hurst Exponents of Noise-like Time Series and the Cantor Set *by Maria C. Mariani, William Kubin, Peter K. Asante, Joe A. Guthrie, and Osei K. Tweneboah*

Financial Return Distributions: Past, Present, and COVID-19 *by Marcin Watorek, Jarosłw Kwapień, and Stanisław Drożdż*

**iii.**  **Correlation, Memory, Dependence and Relatedness**

Continuous Time Random Walk with Correlated Waiting Times. The Crucial Role of Inter-trade Times in Volatility Clustering *by Jarosław Klamut and Tomasz Gubiec*

Understanding Changes in the Topology and Geometry of Financial Market Correlations during a Market Crash *by Peter Tsung-Wen Yen, Kelin Xia, and Siew Ann Cheong*

Understanting the Nature of the Long-Range Memory Phenomenon in Socioeconomic Systems by *Rytis Kazakevičius, Aleksejus Kononovicius, Bronislavas Kaulakys, and Vygintas Gontis*

Are Mobility and COVID-19 Related? A Dynamic Analysis for Portuguese Districts *by Antonio Casa Nova, Paulo Ferreira, Dora Almeida, Andreia Dionisio, and Derick Quintino*

Evolving Network Analysis of S&P500 Components: COVID-19 Influence of Cross-Correlation Network Structure *by Janusz Miśkiewicz and Dorota Bonarska-Kujawska*

Effects of Vaccination Efficacy on Wealth Distribution in Kinetic Epidemic Models *by Emanuele Bernardi, Lorenzo Pareschi, Giuseppe Toscani, and Mattia Zanella*

Asymmetric Relatedness from Partial Correlation *by Carlos Saenz de Pipaon Perez, Andrea Zaccaria, and Tiziana Di Matteo*

**iv.**  **Currency and Cryptocurrency Markets**

Network Analysis of Cross-Correlations on Forex Market during Crises. Globalisation on Forex Market *by Janusz Miśkiewicz*

Neural Networks for Estimating Speculative Attacks Models *by David Alaminios, Fernando Aguilar-Vijande, and José Ramón Sánchez-Serraino*

What Drives Bitcoin? An Approach from Continuous Local Transfer Entropy and Deep Learning Classification Models *by Andrés Garcia-Medina and Toan Luu Duc Huynh*

Cryptocurrency Market Consolidation in 2020–2021 *by Jarosław Kwapień, Marcin Wątorek, and Stanisław Drożdż*

**v.**  **Stock Market**

Analysis of Individual High-Frequency Traders’ Buy–Sell Order Strategy Based on Multivariate Hawkes Process *by Hiroki Watari, Hideki Takyeasu, and Misako Takayasu*

The Stock Market Model with Delayed Information Impact from a Socioeconomic View *by Zhiting Wang, Guiyuan Shi, Mingsheng Shang, and Yuxia Zhang*

A Maximum Entropy Model of Bounded Rational Decision-Making with Prior Beliefs and Market Feedback *by Benjamin Patrick Evans and Mikhail Ptokopenko*

Heterogeneous Criticality in High Frequency Finance: A Phase Transition in Flash Crashes *by Jeremy D. Turiel and Tomasso Aste*

A New Look at Calendar Anomalies: Multifractality and Day-of-the-Week Effect *by Darko Stosic, Dusan Stosic, Irena Vodenska, H. Eugene Stanley, and Tatjana Stosic*

**vi.**  **Company Market**

Learning Your Options: Option-Based Model of Export Readiness and Optimal Export *by Kirill Ilinski*

Multifractal Company Market: An Application to the Stock Market Indices *by Michał Chorowski and Ryszard Kutner*

On the Mortality of Companies *by Peter Richmond and Bertrand M. Roehner*

**vii.**  **Economics vs. Thermodynamics**

Econophysics and the Entropic Foundations of Economics *by J. Barkley Rosser, Jr.*

Energy, Entropy, Constraints, and Creativity in Economic Growth and Crises *by Reiner Kümel and Dietmar Lindenberger*

**viii.**  **Financial Risk**

Aspects of a Phase Transition in High-Dimensional Random Geometry *by Axel Prüser, Imre Kondor, and Andreas Engel*

Optimizing Expected Shortfall under an l_1_ Constraint—An Analytic Approach *by Gábor Papp, Imre Konndor, and Fabio Cacciol*

**ix.**  **Holistic View**

Victory Tax: A Holistic Income Tax System *by Donald J. Jacobs*

Highway Freight Transportation Diversity of Cities Based on Radiation Models *by Li Wang, Jun-Chao Ma, Zhi-Qiang Jiang, Wanfeng Yan, and Wei-Xing Zhou*
